# Editorial: Redox-signaling in neurodegenerative diseases: biomarkers, targets, and therapies

**DOI:** 10.3389/fncel.2023.1198669

**Published:** 2023-04-20

**Authors:** Andreia N. Carvalho, Vasco Branco, Jack van Horssen, Luciano Saso

**Affiliations:** ^1^Research Institute for Medicines (iMed.ULisboa), Faculty of Pharmacy, Universidade de Lisboa, Lisbon, Portugal; ^2^Centro de Investigação Interdisciplinar Egas Moniz, Instituto Universitário Egas Moniz, Caparica, Portugal; ^3^Department of Molecular Cell Biology and Immunology, MS Center Amsterdam, Vrije Universiteit Amsterdam, Amsterdam Neurosciences, Amsterdam UMC Location VUmc, Amsterdam, Netherlands; ^4^Department of Physiology and Pharmacology, Faculty of Pharmacy and Medicine, Sapienza University of Rome, Rome, Italy

**Keywords:** neurodegenerative diseases, post-translational modifications (PTMs), redox-signaling, Nrf2, oxidative stress, antioxidants

Neurodegenerative diseases share common pathological hallmarks such as mitochondrial dysfunction and oxidative stress, which leads to neuronal loss. Redox regulation of key cellular functions is an important signaling mechanism and post-translational modifications (PTMs) are relevant in redox signal transduction and might be instrumental in uncovering pathophysiological mechanisms and identify novel therapeutic targets in neurodegenerative diseases. Promising novel redox-based therapeutics include strategies aimed at enhancing the endogenous antioxidant machinery through activation of the antioxidant master regulator nuclear factor erythroid 2-related factor 2 (Nrf2) or modulation of reactive oxygen species (ROS) production by nicotinamide adenine dinucleotide phosphate (NADPH) oxidases (NOXs) inhibitors (Carvalho et al., 2017).

This Research Topic focuses on redox signaling, particularly Nrf2-driven mechanisms, and aims to provide novel insights into redox regulation involving PTMs in the pathophysiology of neurodegenerative diseases.

Seminotti et al. review the current knowledge on the dysregulation of the Nrf2 pathway in the pathophysiology of inherited metabolic disorders (IMDs), rare genetic conditions affecting predominantly the central nervous system (CNS). The authors revise the critical role of Nrf2 signaling in IMDs and discuss the beneficial effects of Nrf2 activators as potential therapeutic options. Nrf2 pathway dysfunction has been associated with complex metabolic disorders, such as diabetes. This highlights the importance of adequate Nrf2 function in preventing the onset of diabetes and impaired Nrf2 function as a critical mediator of diabetes progression (Dodson et al., 2022). In type II diabetes chronic hyperglycaemia is associated to increased pro-inflammatory signaling, mitochondrial dysfunction, and impaired autophagy, contributing to neurodegeneration. In this issue Dedert et al. investigated the role of progranulin in preserving the autophagic flux and mitochondrial function in neurons under hyperglycaemic conditions. Progranulin treatment upon high-glucose stress conditions, led to the activation of glycogen synthase kinase 3β (GSK3β). GSK3β is a known endogenous negative regulator of Nrf2 activity (Rada et al., 2011), which suggests that modulation of Nrf2 might constitute an alternative mechanism for neuronal autophagic flux regulation under high-glucose stress conditions.

Hyperglycaemia is a risk factor for several neurodegenerative diseases, such as Alzheimer's and Parkinson's disease (Madhusudhanan et al., 2020). Saha et al. reviewed the potential role of Nrf2 as a key target for therapeutic intervention in these neurodegenerative conditions due to its dual anti-inflammatory and antioxidant functions. They suggest that Nrf2 activation could be a novel therapeutic approach since it serves as an integration hub for inflammatory and oxidative signals.

In fact, the protective role of Nrf2 in the CNS extends beyond neurodegenerative conditions. In an original research article Yang et al. demonstrated that Nrf2 protects sensory hair cells from gentamicin-induced damage and ototoxicity and eventual hearing loss, *via* induction of its downstream target heme oxygenase 1. This protective effect was absent in the presence of Nrf2 or heme oxygenase inhibitors.

Additional articles in this topic issue focus on the role of ROS in stroke, a devastating medical condition classified as both cardiovascular and neurological disorder. Liu X. et al. reported that serum levels of 4-hydroxynonenal, a product of lipid peroxidation frequently used as a biomarker of oxidative stress (Carvalho et al., 2017), are related with increased risk of recurrence of patients with primary cerebral infarction, establishing serum 4-hydroxynonenal levels as an independent risk factor that may become a new target for prevention of stroke recurrence. Lõhelaid et al. discuss potential strategies to boost endogenous protective pathways to improve stroke outcomes. One such strategy might be promoting the unfolded protein response, an evolutionary conserved adaptive stress response. The authors focus on mesencephalic astrocyte-derived neurotrophic factor (MANF) due to its putative pro-survival effects in several disease models such as diabetes, neurodegeneration and stroke, and perform a systematic comparative analysis of MANF and X-box binding protein 1, another important effector of the unfolded protein response.

In the CNS, ROS are important regulatory signals for synaptic plasticity. Sobrido-Cameán et al. reported that activity regulated growth of motoneurons at the neuromuscular junction is mediated by ROS sources, namely NOXs. The authors found fundamental differences between pre- and post-synaptic responses and showed that specific aquaporins are mediators of NOXs-dependent changes in pre-synaptic motoneuron growth.

Another regulatory layer of post-synaptic signaling that is redox-mediated is the modulation of chloride ions concentration by nitric oxide, that was investigated by Rodriguez et al. in their paper and that determines the inhibitory or excitatory nature of GABAergic and glycinergic synapses. The authors suggested that reduced expression of TMEM16A, a calcium activated chloride channel that is also expressed in neurons, impairs the nitric oxide-dependent chloride release in retinal amacrine cells.

PTMs might be a critical phenomenon in redox mediated signaling. In this Topic issue, Liu D. et al. reviewed N6-methyladenosine (m6A) modification as a potential regulatory mechanism in spinal cord injury, a traumatic injury that severely affects the CNS. Changes in m6A levels are associated with alterations in the spinal cord microenvironment upon injury, such as ischemia, inflammation and apoptosis. The latest progresses made in the regulation of m6A modification and its relationship with pathological mechanisms involved in spinal cord injury are discussed.

Coenzyme A (CoA), a key metabolite in cellular bioenergetics and neurotransmitter biosynthesis, has recently been attributed a new antioxidant function involving covalent protein modification, CoAlation, which was reported to modulate protein activity and protects cysteine residues from overoxidation (Tsuchiya et al., 2017). Here, Lashley et al. reported extensive anti-CoA immunostaining in brain tissue of various neurodegenerative diseases. The authors show that Tau is covalently modified by CoAlation, with the modification mapped by mass spectrometry to a conserved cysteine residue in the microtubule binding region, suggesting that this PTM might play an important role in protecting redox-sensitive tau cysteine from irreversible overoxidation. CoAlation was additionally shown to consistently co-localize with tau-positive neurofibrillary tangles in AD brains, highlighting the relevance of this PTM in AD.

Overall, the present collection of articles contributes to the identification of mechanisms of redox regulation in neurophysiology and neuropathology and deepen our understanding on the role of reactive species in neurodegenerative diseases, with particular emphasis on Nrf2-driven mechanisms and redox-regulation involving post-translational modifications.

## Author contributions

This editorial was led by AC who wrote the draft of the manuscript. AC, VB, JH, and LS critically revised the manuscript for important intellectual content. All authors provided approval for publication of the content.
